# CRYPTOCOCCAL meningitis in a HIV negative newly diagnosed diabetic patient: a CASE report

**DOI:** 10.1186/s12879-018-3625-4

**Published:** 2019-01-03

**Authors:** Odhiambo Henry Owuor, Patrick Chege

**Affiliations:** School of Medicine, Department of Family Medicine, Moi University, P. O. Box, Eldoret, 3900-30100 Kenya

**Keywords:** Cryptococcal meningitis, Diabetes, Fluconazole, HIV negative

## Abstract

**Background:**

This case report emphasizes the need to recognize cryptococcus as a possible cause of meningitis in non-HIV patients in Sub-Saharan Africa and to highlight the possibility of grave outcomes due to the paradoxical immune response in diabetic patients with cryptococcus meningitis. It also highlights the need for widespread availability of amphotericin-B and flucytosine in hospitals in Sub-Saharan Africa.

**Case presentation:**

A 27 year old African lady was admitted with generalized tonic clonic seizures lasting 5 to 10 min. These seizures were preceded by severe frontal headaches radiating to the occiput and neck and associated with chills, photophobia and loss of consciousness. She was tachycardic and had tongue bites on the lateral aspects of her tongue. Kernig’s and Brudzinski’s signs were positive. India ink was positive on two cerebrospinal fluid (CSF) samples. She had hyperglycemia and glucosuria as well. She was diagnosed with cryptococcal meningitis in diabetes and had a remarkable response to fluconazole monotherapy. She went home on maintenance dose of fluconazole having made full recovery. and is currently on prophylactic doses of fluconazole.

**Conclusions:**

With the rising prevalence of diabetes in Sub-Saharan Africa, coupled with the low levels of adequate glucose control, cryptococcal meningitis should be considered in the differential diagnosis for diabetic patients presenting with chronic headache, fever and neurologic deficits.

## Background

*Cryptococcus neoformans* is an opportunistic fungus that causes infection of the central nervous system in those who are immunocompromised, particularly in human immunodeficiency virus (HIV) patients [1]. It infrequently infects HIV-negative persons such as post-organ transplant patients and those with diabetes mellitus, liver cirrhosis and alcoholism [1]. Inadequate glucose control in diabetic patients increases their risk to infections due to numerous factors especially the glucose-rich blood which is media for micro-organism growth [2–4]. Remarkably, diabetic patients mount dramatic immune responses to cryptococcus that are paradoxical to their immunosuppressive state. Studies have shown that optimal control of blood sugar levels in diabetic patients in Sub-Saharan Africa is very low [5–7]. This case report emphasizes the need to recognize cryptococcus as a possible cause of meningitis in non-HIV patients in Sub-Saharan Africa and to highlight the possibility of grave outcomes due to the paradoxical immune response in diabetic patients with cryptococcal meningitis. The case report also highlights the need for widespread availability of amphotericin-B and flucytosine in hospitals in Sub-Saharan Africa.

## Case presentation

A 27 year old African lady, from Bungoma County in Western Kenya, was admitted with generalized tonic clonic seizures lasting 5 to 10 min. This was associated with loss of consciousness for 10–20 min and left sided hemiparesis that lasted nearly 30 min after the convulsion. These symptoms had lasted one day but had been preceded by severe frontal headache radiating to the occiput and neck, chills, and photophobia for three days. Her past medical history was significant for peptic ulcer disease and allergic rhino-sinusitis for which she uses omeprazole, antacid and celestamine intermittently. Social history is notable for a diabetic twin sister who had been on insulin. The twin sister had died of pneumonia three days to the patient’s admission. The patient’s mother died ten years ago due to complications associated with diabetes. She is separated from her husband but she stays with her six year old daughter. The patient runs a small pharmacy business. She had travelled with her twin sister to a funeral before they fell sick.

The patient had been sickly for a month before this admission. She had had severe frontal headaches associated with chills and photophobia. She had also complained of frequency of micturition and increased intake of water. There was no weight loss. She was treated with artemether-lumefantrine (AL) at a health center for malaria (blood slide was positive for *P. falciparum*). There was improvement for two weeks. However, the headache and photophobia recurred while polyuria and polydypsia persisted. She sought medical attention in a different health center where she was diagnosed with severe malaria and diabetes. Metformin was instituted in addition to quinine and AL. She developed the convulsions on the third day of treatment and was brought to our facility.

### Clinical findings

Her vital signs was notable for tachycardia (pulse was 112 beats/min). Her blood pressure (BP) was 114/75 mmHg, saturations 93%, respiratory rate was 15 cycles/min, and temperature was 36.7 °C. She had bites on the lateral aspects of her tongue, an equivocal neck and positive Kernig’s and Brudzinski’s signs. Reflexes and remainder of her physical exam was normal.

### Timeline

**Table:** 

Date	Relevant Patient Data
December 3, 2017 (day 1/admission date)	Patient admitted with:
	Severe headaches;
	Convulsions;
	Difficulty swallowing;
	Polyuria;
	Polydipsia;
	Tachycardia;
	Positive Kernig’s;
	Right upper quadrant (RUQ) tenderness;
	Diagnosed with bacterial meningitis in a diabetic; with differentials of cerebral malaria, brain abscess;
	Labs
	1. FBS: 19.1 mmol/l Daily FBS
	2. PITC: non-reactive;
	3. LP for CSF analysis: biochemistry, microscopy, India ink, ZN staining.
	4. ESR: 87 mm/hr.;
	5. BS for MPS: 20 parasites per 200 wbcs;
	6. FHG, UECs, CT scan: patient did not do these because of lack of funds;
	7. Urinalysis: ph = 7.0; glucose +++; blood ++; SG 1.010; ketones nil; deposit: nothing seen.
	8. Abdominal ultrasound: normal abdominal scan, no renal or splenic abscess.
	Treatment:
	1. Diazepam 10 mg PRN
	2. Artesunate: 180 mg at 0, 12 and 24 h;
	3. Ceftriaxone 2 g BD for 10 days;
	4. Paracetamol 1 g tid
	5. Metformin 500 mg bd
	6. Dentogel cream apply QID on the oral lesions for 5 days.
	7. IV fluids 4 l of normal saline in 24 h
Day 2	Patient had four convulsions last night. Another which was partial occurs during the round.
	On examination: left sided paresis noted. All vitals including heart rate were now normal.
	Results:
	1. CSF: turbid; 60 cells/mm^3^; Pandy test negative; India ink positive; gram stain: no organism isolated; ZN negative; VDRL: negative.
	2. FBS = 17.4 mmol/l;
	3. I.V phenytoin 100 mg BD
	4. Fluconazole 1200 mg od for 2 weeks, then 400 mg od for 10 weeks; then 200 mg od for 6 months.
	5. Daily FBS; do UECs, FHG, CT scan of the head, TSH
	6. Continue with ceftriaxone, metformin, PCM, dentogel, and artesunate,
	7. Monitor for seizure occurrence and chart
Day 3	Reports improvement with no seizures reported; patient is now able to feed, has mild headaches.
	FBS not reported;
	Vitals normal (BP = 112/72 mmHg, pulse 97 bpm, SPO2 = 94%);
	Neck is soft and Kernig’s negative.
	Plan: continue with fluconazole, ceftriaxone, phenytoin, paracetamol, aremether-lumefantrine
Day 4–6	Reports some neck pains and headaches;
	On examination, patient is in fair general condition, vitals are normal;
	Neck is soft and Kernig’s negative;
	FBS oscillates between 13 and 14 mmol/l;
	Plan: continue with medication, do nutritional counseling because of diabetes;
Day 7	Patient complaints of frontal headache, itchiness on areas that had strapping used to secure IV lines, and gluteal itchiness
	FBS = 13.7 mmol/l;
	Patient is once again sick looking, with normal vitals (BP = 116/79 mmHg; SPO_2_ = 95%; pulse = 85)
	Neck is stiff and tongue lesions are still present on the edge of the tongue;
	Pruritic pustules on the areas with strapping, buttock has no eruptions;
	Plan:
	1. I.M diclofenac 75 mg PRN;
	2. hydrocortisone cream to apply bd;
	3. soluble insulin 5 IU tid;
	4. continue antimeningitics and phenytoin;
	5. do daily pre-dinner RBS and FBS.
Day 8	Patient still complaining of severe headache despite administration of three doses of diclofenac; the tongue ulcer is still painful and she is still itching;
	Patient is sick looking but with normal vitals;
	She still has the stiff neck, rashes and the tongue ulcers;
	Plan:
	1. 4mls of clear CSF tapped and sent for microscopy: India ink positive;
	2. Continue with the medication from the previous day.
Day 9	The headaches have improved and her vitals are normal;
	FBS = 20.9 mmol/l;
	Plan:
	1. stop the insulin;
	2. increase metformin to 1 g bd;
	3. continue with other medication.
Day 10–13	Patient reports improvement and her vitals are normal.
	RBS ranged between 8.0 and 13.1 mmol/l.
	Plan:
	1. continue with management as per treatment sheet i.e. ceftriaxone, fluconazole, metformin, glibenclamide, hydrocortisone cream and dentogel.
	2. Do serial CSF tapping if headaches are persistent.
Day 14	Patient has no complaints today.
	FBS = 8 mmol/l.
	Plan:
	1. Allow home on metformin 750 mg bd;
	2. Fluconazole 800 mg od for two weeks then 400 mg for 10 weeks;
	3. Dentogel, hydrocortisone cream, and omeprazole.
	4. To come again on 12th January 2018.
1st clinic visit (January 11, 2018)	Patient is doing well except for a flare up of her allergic rhino-sinusitis. Blood sugar was 5.8 mmol/l and a repeat lumber puncture was negative on India ink test.
	The need for dietary prudence and drug adherence was emphasized. She was then given fluconazole 800 mg od and asked to continue with metformin 750 mg bd, cetirizine 10 mg prn, omeprazole 20 mg bd and hydrocortisone.
2nd clinic visit (January 25, 2018)	Patient diagnosed with pneumonia;
	RBS = 8.2 mmol/l; BS for MPS was negative; CSF for India ink was negative for Cryptococcus;
	Plan:
	-septrin 960 mg bd for one week;
	-paracetamol 1 g tid for 5 days;
	-omeprazole 20 mg 1 h before supper for a month;
	-fluconazole 800 mg od for a month;
	-metformin 500 mg bd for a month;

### Diagnostic assessment

Her initial lab results showed 20 malaria parasites/200WBCs on a blood slide, a random blood sugar of 19.1 mmol/l, HIV non-reactive, ESR 87 mm/hr., cerebro-spinal fluid (CSF): turbid, 60 cells/mm^3^, pandy test negative, Indian ink positive, gram stain: no organism isolated, Ziehl–Neelsen stain (ZN) negative, venereal disease research laboratory (VDRL): negative; full hemogram (FHG), urea, electrolytes and creatinine (UEC) & CT scan: patient did not do these because of lack of funds; urinalysis: ph = 7.0, glucose +++, blood ++, SG 1.010, ketones nil, deposit: nothing seen; abdominal ultrasound: normal abdominal scan, no renal or splenic abscess. Fundoscopy neither revealed malarial retinopathy nor papilledema. We could not get a CT scan, urea/electrolytes/creatinine and a full hemogram/complete blood count done due to the patient’s financial status.

### Intervention

The patient was started on diazepam 10 mg PRN, artesunate: 180 mg at 0, 12 and 24 h, ceftriaxone 2 g BD for 10 days, paracetamol 1 g tid and metformin 500 mg bd. Fluconazole 1200 mg od for 2 weeks was initiated due to the unavailability of amphotericin B and flucytosine. Convulsions ceased on the third day. There was improvement over the next few days but on the eighth day there was severe headache, neck stiffness and positive Kernigs sign. Serial tapping was done to relieve the headaches.

### Follow-up and outcomes

After 2 weeks and with marked improvement, the patient was allowed home on metformin 750 mg bd, fluconazole 800 mg od for ten weeks. A repeat CSF analysis was negative for Indian ink after two weeks of treatment. She has been free of symptoms since she was allowed home. Metformin dose was reduced to 500 mg bd because her blood sugar was controlled and ranging between 5 and 8 mmol/l. She was counseled on lifestyle modification, particularly on diet and exercise to enhance control of diabetes. Other findings during her follow up are as outlined in the ‘Timeline’ table.

## Discussion and conclusions

*Cryptococcus neoformans* is an opportunistic fungus that causes infection of the central nervous system in those who are immunocompromised, particularly in human immunodeficiency virus (HIV) patients [1]. It is rarely reported as a cause of meningitis in HIV seronegative patients in Sub-Saharan Africa. However, it may infect persons with other immunosuppressed systems like patients on chronic glucocorticoid use, malignancy, sarcoidosis and liver failure [1]. Sub-Saharan Africa is experiencing an increasing prevalence of diabetes [2]. Of note is that low levels of adequate glucose control are achieved in diabetic patients who are on any form of treatment [3, 4]. This is bound to increase the occurrence of cryptococcal meningitis in non HIV non transplant patients, who are predominantly diabetic patients.

*C. neoformans* is acquired through inhalation. From the respiratory tract it causes disseminated disease with a propensity for the central nervous system (neourotopism). Sometimes it may spread to the bone, prostate, skin, eyes and liver [1, 8]. Cryptococcal meningitis can present as fever, malaise, headache, vomiting, meningeal signs, seizures and cranial nerve palsies [8, 9]. These features tend to be sub-acute with the headache evolving over 2–4 weeks [10].

Diabetic patients with poorly controlled blood sugars are more prone to infections since the glucose-rich blood serves as an excellent media for growth. Cryptococcal infections in these patients are severe. Hyperglycemia causes impaired granulocyte chemotaxis, phagocytosis and deficient cell-mediated immunity, which is the most important host response to cryptococcal infection. Elevated serum levels of TNF-α and interleukin-6 in patients with diabetes let them mount an exaggerated immune response to *C. neoformans* [5–7]. This milieu is a recipe for infections to lead to fatal outcomes as reported by Kushawaha and colleagues in their case report [1].

This lady was on the alternative regimen for treating cryptococcal meningitis in adults [11]. In the induction phase, she was given fluconazole 1200 mg od for two weeks. This was followed by 800 mg of fluconazole for the consolidation phase of 8 weeks and 400 mg for the maintenance phase. CSF sterilization was confirmed in the fourth week and the patient has remained asymptomatic. This is in contrast to the recommended combination of amphotericin B and flucytosine for the induction phase that provides greater fungicidal activity, early CSF sterilization, lower rates of treatment failure and lower mortality [12–15].

Due to the exaggerated immune response, some scholars offer that anti-inflammatory medication like corticosteroids may have an adjunctive role in the management of cryptococcal meningitis in diabetic patients [1, 16]. Lack of a CT scan, and inability to estimate serum cryptococcal antigen or to do a CSF culture limited the work up of this patient.

In conclusion, this case demonstrates that with the rising prevalence of diabetes in Sub-Saharan Africa, just like in China, cryptococcal meningitis should be considered in the differential diagnosis for diabetic patients presenting with chronic headache accompanied with fever and neurologic deficits [17]. It is important to note that presentations may be dramatic and the outcomes grave due to the paradoxically exaggerated immune response to *C. neoformans* [18]. The response to fluconazole was satisfactory in this patient. However, effort should be made to avail the more effective combination of amphotericin B and flucytosine in resource poor setting. Modalities like laboratory services to monitor any toxicities should also be availed. Otherwise researchers should endeavor to come up with cheaper, effective but less toxic formulations. A co-positive state with malarial parasites may not preclude the diagnosis, and treatment thereof, of cryptococcal meningitis especially in a malaria-endemic zone. A high index of suspicion may be extremely rewarding in such a presentation.

## Abbreviations

AL: Artemether-lumefantrine
BD: Twice a day
BP: Blood pressure
BS for MPS: Blood slide for malaria parasites
CSF: Cerebro-spinal fluid
ESR: Erythrocyte sedimentation rate
FBS: Fasting blood sugar
FHG: Full hemogram
HIV: Human immunodeficiency virus
IU: International units
IV: Intravenous
LP: Lumber puncture
OD: Once a day
PCM: Paracetamol
PITC: Provider initiated testing and counseling for HIV
PRN: When necessary
QID: Four times a day
RBS: Random blood sugar
RUQ: Right upper quadrant
SPO_2_: Peripheral capillary oxygen saturations
TID: Three times a day
UECs: Urea, electrolytes and creatinine
VDRL: Venereal disease research laboratory
ZN: Ziehl–Neelsen
